# The Clinical Outcomes and Toxicities of Induction Chemotherapy Followed by Concurrent Chemoradiotherapy Plus Adjuvant Chemotherapy in Locoregionally Advanced Nasopharyngeal Carcinoma

**DOI:** 10.3389/fonc.2020.619625

**Published:** 2021-02-26

**Authors:** Rui Zou, Jing-Jing Yuan, Qiang Li, Jian-Wu Ding, Bing Liao, Zi-Wei Tu, Rong-Huan Hu, Dan Gong, Jia-Li Hu, Lei Zeng

**Affiliations:** ^1^ Department of Oncology, The Second Affiliated Hospital of Nanchang University, Nanchang, China; ^2^ Jiangxi Key Laboratory of Clinical Translational Cancer Research, Nanchang, China; ^3^ Medical College of Nanchang University, Nanchang, China; ^4^ Department of Lymphatic Hematologic Oncology, Jiangxi Cancer Hospital of Nanchang University, Nanchang, China; ^5^ Department of Otorhinolaryngology Head and Neck Surgery, The Second Affiliated Hospital of Nanchang University, Nanchang, China; ^6^ NHC Key Laboratory of Personalized Diagnosis and Treatment of Nasopharyngeal Carcinoma, Jiangxi Cancer Hospital of Nanchang University, Nanchang, China

**Keywords:** induction chemotherapy, concurrent chemotherapy, adjuvant chemotherapy, intensity-modulated radiotherapy, nasopharyngeal carcinoma

## Abstract

**Purpose:**

To analyze the outcomes and toxicities of induction chemotherapy (ICT) followed by concurrent chemoradiotherapy (CCRT) plus adjuvant chemotherapy (ACT) in patients with locoregionally advanced nasopharyngeal carcinoma (LA-NPC).

**Methods:**

Retrospective analysis of 163 patients with LA-NPC referred from August 2015 to December 2018 was carried out. All patients underwent platinum-based ICT followed by CCRT plus ACT.

**Results:**

The median follow-up time was 40 months, ranging from 5 to 69 months. The 3-year disease-free survival (DFS), overall survival (OS), locoregional recurrence-free survival (LRRFS), and distant metastasis-free survival (DMFS) rates were 80.8, 90.0, 91.6, and 87.4%, respectively. The most frequent acute grade 3/4 adverse events were leukopenia (66.8%), neutropenia (55.8%), mucositis (41.1%), thrombocytopenia (27.0%), and anemia (14.7%).

**Conclusion:**

ICT followed by CCRT plus ACT did not seemingly enhance DFS and OS in LA-NPC patients compared to the addition of ICT to CCRT (historical controls). In contrast, ICT followed by CCRT plus ACT had more acute adverse events than ICT followed by CCRT. Longer-term clinical studies are required to examine the treatment outcomes and late toxicities.

## Introduction

The location of nasopharyngeal cavity is deep and hidden, and hence, the lesions are notoriously difficult to detect at an early stage. At presentation, more than 70% of nasopharyngeal cancer patients have advanced disease due to late diagnosis (1). The treatment for locoregionally advanced nasopharyngeal carcinoma (LA-NPC) usually involves a combination of concurrent chemoradiotherapy (CCRT) with or without induction chemotherapy (ICT) or adjuvant chemotherapy (ACT) (2).

The landmark Intergroup-0099 randomized trial was the first to demonstrate that concurrent treatment of CCRT and ACT could increase the 3-year overall survival (OS) rate by 31% compared to radiotherapy alone group (3). Thenceforth, this regimen is deemed as a standard-of-care for patients with LA-NPC. A recent study has compared the outcomes of 508 stage III-IVB patients who had undergone CCRT with or without ACT, and found that the survival endpoints, such as failure-free survival (FFS) and OS, were not markedly improved in the ACT arm (4). However, a meta-analysis conducted by MAC-NPC collaborative group has shown that the use of chemotherapy could be beneficial for improving the survival endpoints (e.g., cancer mortality, distant control, locoregional control, and progression-free survival), and based on the timing of chemotherapy, CCRT plus ACT was most favorable compared to CCRT alone, ICT alone, and ACT alone (5). Thus far, CCRT plus ACT is still considered as 2A recommendation in the National Comprehensive Cancer Network (NCCN) 2020 guidelines for treating head and neck tumors.

Compared to ACT, ICT provides the advantages of improved tolerability and early eradication of micrometastases. Therefore, ICT followed by CCRT can serve as a potential treatment strategy for LA-NPC patients receiving intensity-modulated radiation therapy (IMRT). In recent years, two large multicenter phase 3 trials reported that ICT markedly enhanced FFS, OS, and distant failure-free survival in LA-NPC after the addition of CCRT (6–8). A pooled analysis of individual patient data from four randomized trials in endemic areas was carried out, and found that the combination of ICT and CCRT remarkably improved OS (9). To summarize the existing findings, the 2018 NCCN guidelines upgraded the evidence for IC plus CCRT from level 3 to level 2A, which is similar to that of CCRT plus ACT.

He et al (10). have reported the efficacy and safety of ICT followed by IMRT plus ACT. The 3-year estimated rates of locoregional control, OS, and metastasis-free survival were 94.4, 87.7, and 86.2%, respectively, and the toxicity profile is acceptable. However, it remains unclear whether ICT followed by CCRT plus ACT can improve the outcomes of LA-NPC patients. This research aimed to analyze the outcomes and toxicity profiles of ICT followed by CCRT plus AC in patients with LA-NPC.

## Subjects and Methods

### Patients and Pretreatment Assessment

The histologically confirmed (WHO type II/III), previously untreated, advanced (stage III-IVB using the seventh edition of AJCC/UICC staging system) LA-NPC patients were recruited from August 2015 to December 2018. Other inclusion criteria included 18–70 years old; Karnofsky scale ≥70; received IC followed by CCRT and ACT; no evidence of distant metastases; normal renal, hepatic, and hematologic function. Based on these criteria, a total of 163 LA-NPC patients were included in the study group, and their baseline characteristics are presented in **Table 1**. The initial evaluation included chest and abdominal CT scans, bone scintigraphy, nasopharyngoscopy, and nasopharyngeal-neck magnetic resonance imaging (MRI) scan.

**Table 1:** Baseline characteristic of the 163 LA-NPC patients.

	Total	
	n	%
Sex Male Female	117 46	71.8 28.2
Age (years) ≤50 >50	80 83	49.1 50.9
T category T1 T2 T3 T4	3 9 90 61	1.9 5.5 55.2 37.4
N category N0 N1 N2 N3	8 80 58 17	4.9 49.1 35.6 10.4
Stage III IVA IVB	91 55 17	55.8 33.7 10.5

### Radiation Therapy

All LA-NPC patients were exposed to IMRT (6 MV photons). Target volume delineation was performed in accordance with the treatment protocol of Sun Yat-Sen University Cancer Center, which adhered to the reports 50 and 62 of the International Commission on Radiation Units and Measurements Reports (ICRU) (11–13). After delineating the tumor targets based on the patient immobilization and target localization, clinical target volumes, and planning target volumes were obtained using a simultaneous integrated boost strategy. The prescribed doses to the planning target volumes of the primary nasopharyngeal gross tumor volume, the first clinical target volume, the second clinical target volume, and the cervical metastatic lymph node gross tumor volume were 66–70, 60, 54–56, and 64–66 Gy, respectively. No patient failed to complete the radiotherapy.

### Chemotherapy

The patients were intravenously administered with ACT consisting of TP (paclitaxel 135 mg/m^2^ on the first day and cisplatin/nedaplatin 25 mg/m^2^/day on the first 3 days), DP (docetaxel 75 mg/m^2^ on the first day and cisplatin/nedaplatin 25 mg/m^2^/day on the first 3 days), TPF (cisplatin/docetaxel 60 mg/m^2^ on the first day and fluorouracil 600 mg/m^2^/day as a continuous 120-h infusion on days 1–5), and GP (gemcitabine 1000 mg/m^2^/day on days 1 and 8, cisplatin/nedaplatin 25 mg/m^2^/day on the first 3 days). Cisplatin/nedaplatin-based chemotherapy began concurrently on weeks 1, 4, or 7 of radiation therapy. **Table 2** shows the detail compliance of ICT, CCRT, and ACT. Only sixty patients received at least 2 cycles of ICT, CCRT, and ACT each. Nine patients receiving ICT had dose reductions. Thirty-four patients receiving ACT had dose reductions.

**Table 2:** Compliance of chemotherapy for 163 patients with LA-NPC.

Chemotherapy	Total	
	No.	%
ICT cycles 1 cycle 2 cycles 3 cycles 4 cycles	1 50 109 3	0.6 30.7 66.9 1.8
CCRT cycles 1 cycle 2 cycles 3 cycles	89 61 13	54.6 37.4 8.0
ACT cycles 1 cycle 2 cycles 3 cycles 4 cycles	45 43 74 1	27.6 26.4 45.4 0.6
ICT or ACT regimen TP DP TPF GP	19 124 15 5	11.7 76.1 9.2 3.0

### Follow-up

After completing radiotherapy, all patients were followed-up every 1–3 months for 2 years, every 6 months for the next 3 years, and every 12 months thereafter. To assess the disease status and treatment toxicity, physical examination, abdominal ultrasonography, chest radiography, and head/neck MRI scans were performed during the follow-up periods. A whole-body bone scintigraphy was carried out if necessary.

### Statistics

SPSS 19.0 was employed for statistical analyses. The Kaplan-Meier curves were constructed to estimate disease-free survival (DFS), distant metastasis-free survival (DMFS), locoregional recurrence-free survival (LRRFS), and OS rates. Multivariable analysis was carried out by using the Cox proportional hazards model with forward and backward stepwise regression, in order to identify potential variables associated with DFS, DMFS, LRRFS, and OS. Covariates included gender (female *versus* male), age (>50 years *versus ≤*50 years), performance status (0 *versus* 1), N category (N2 *versus* N0-1; N3 *versus* N0-1), and T category (T3 *versus* T1-2; T4 *versus* T1-2) were selected. The two-tailed significance level was set to 0.05.

## Results

### Failure Patterns and Survival Analysis

**Figure 1:**
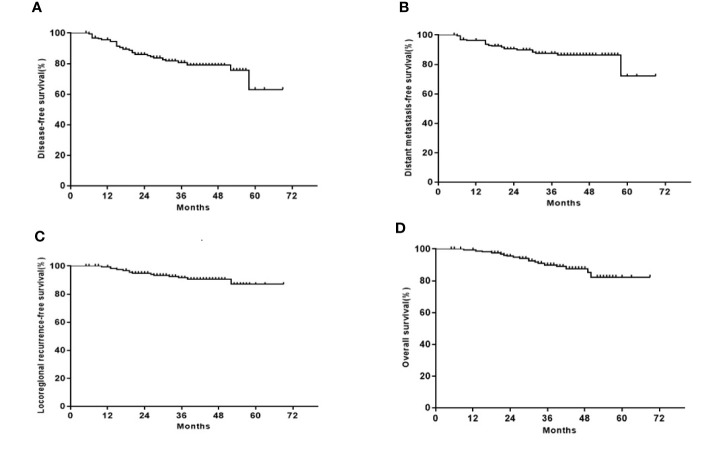
Kaplan–Meier curves of the 163 patients with LA-NPC. **(A)** DFS; **(B)** OS; **(C)** LRRFS; **(D)** DMFS.

After a median follow-up of 40 months (ranging 5 to 69 months), treatment failures were observed in 33 patients. Among them, 21 had distant metastases, 11 had primary NPC recurrence, and 2 had both regional nodal and primary NPC recurrences. Of the 18 patients who had died, 9 had locally recurrent disease, 8 had distant metastases, and 1 had nasopharyngeal hemorrhage. The 3-year DFS, OS, LRRFS, and DMFS rates were 80.8, 90.0, 91.6, and 87.4%, respectively (**Figure 1**).

### Subgroup Analyses and Prognostic Factors

**Figure 2:**
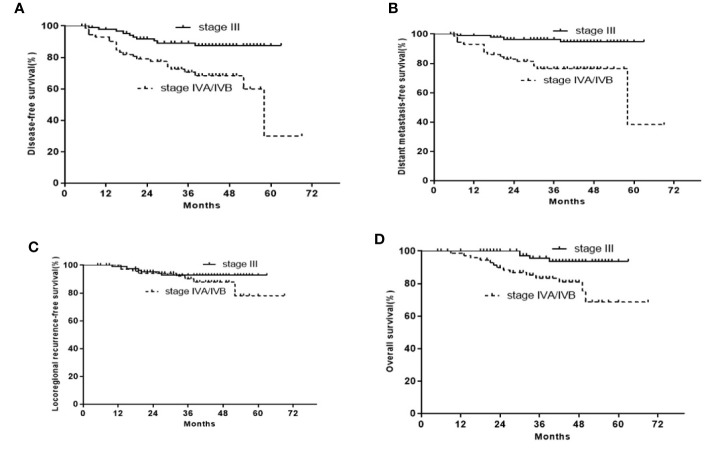
Kaplan-Meier curves of the 163 stage III or IVA/IVB LA-NPC patients. **(A)** DFS; **(B)** OS; **(C)** LRRFS; **(D)** DFMS.

The 3-year DFS, OS, LRRFS, and DMFS rates were 89.2, 95.7, 92.7, and 96.5% for stage III LA-NPC, and 70.6, 83.3, 90.2, and 76.4% for stage IVA/IVB LA-NPC, respectively (**Figure 2**). The 3-year DFS rates were 100, 87.4, 72.4, and 69.5%, respectively for stage N0-3 LA-NPC patients. The 3-year OS rates were 100, 89.8, 89.6, and 87.8%, respectively for stage N0-3 LA-NPC patients. The 3-year DMFS rates were 100, 93.4, 81.3, and 75.5%, respectively for stage N0-3 patients. In the multivariate analysis of all 163 patients, T category was identified as an independent prognostic factor for DFS and DMFS; while N category was independently associated with DFS, DMFS, and OS. However, no clinical factors were independently correlated with LRRFS (**Table 3**).

**Table 3:** Significant prognostic factors for the survival rates of LA-NPC patients.

End point/factors	HR (95% CI)	P-value
DFS T4 *vs.* T1,2 N2 *vs.* N0,1 N3 *vs.* N0,1	3.194 (1.554–6.562) 2.999 (1.348–6.672) 4.219 (1.581–11.257)	0.002 0.007 0.004
DMFS T4 *vs.* T1,2 N2 *vs.* N0,1 N3 *vs.* N0,1	6.347 (2.290–17.593) 3.214 (1.158–8.917) 5.248 (1.564–17.613)	<0.001 0.025 0.007
LRRFS T3 *vs.* T1,2 T4 *vs.* T1,2 N2 *vs.* N0,1 N3 *vs.* N0,1	2.164 (0.230–20.316) 2.996 (0.285–31.457) 2.266 (0.682–7.528) 2.306 (0.405–13.145)	0.499 0.360 0.182 0.347
OS N2 *vs.* N0,1 N3 *vs.* N0,1	2.956 (1.004–8.709) 3.470 (0.654–18.417)	0.049 0.144

### Treatment Toxicities

Over the entire treatment course, 128 of 163 patients (78.5%) had acute grade 3/4 adverse events. Leukopenia was the most frequent adverse event (110 in 163 patients [67.5%]), followed by neutropenia (91 patients [55.8%]) and mucositis (67 patients [41.1%]). Besides, the incidence rate of late grade 1/2 toxicities was 79.1% (129/163), and 6.7% (11 in 163) patients had one or more late grade 3/4 toxicities. The acute and late toxicities of LA-NPC treatment are listed in **Table 4**.

**Table 4:** Treatment toxicities of the 163 patients with LA-NPC.

Event	Number of patients (n = 163)	
	Grade 1 or 2 (%)	Grade 3 or 4 (%)
Any acute adverse event Leukopenia Neutropenia Anemia Thrombocytopenia Hepatic Creatinine Nausea Vomiting Xerostomia Mucositis Dermatitis Any late adverse event Temporal lobe injury Neck fibrosis Trismus Xerostomia Dysphagia Hearing impairment	35 (21.5) 51 (31.3) 62 (38.0) 130 (79.8) 84 (51.5) 78 (47.9) 11 (6.7) 136 (83.4) 76 (46.6) 121 (74.2) 96 (58.9) 101 (62.0) 129 (79.1) 6 (3.7) 47 (28.8) 7 (4.3) 115 (70.6) 3 (1.8) 40 (24.5)	128 (78.5) 110 (67.5) 91 (55.8) 24 (14.7) 44 (27.0) 2 (1.2) 0 10 (6.1) 9 (5.5) 7 (4.3) 67 (41.1) 4 (2.5) 11 (6.7) 0 0 0 6 (3.7) 0 11 (6.7)

## Discussion

Meta-analysis and clinical trials showed that the addition of ICT to CCRT demonstrated a remarkable enhancement on both tumor control and survival in patients with LA-NPC (6–8, 14, 15). Although the effects of ACT on OS and RFS remain controversial, CCRT plus ACT is still an option for LA-NPC patients according to the NCCN guidelines for head and neck cancers version 2020. However, it remains unclarified whether an increase in chemotherapy dose intensities can enhance the outcomes of LA-NPC patients.

We were first to report the outcomes of ICT followed by CCRT plus ACT in LA-NPC patients. In this retrospective study, the 3-year DFS, OS, and DMFS rates were 80.8, 90.0, and 87.4%, respectively. Overall, 128 of 163 patients (78.5%) had acute grade 3/4 adverse events. Leukopenia was the most frequent event (110 in 163 patients [67.5%]), followed by neutropenia (91 patients [55.8%]) and mucositis (67 patients [41.1%]). Sun et al (6). reported that the 3-year recurrence-free survival (RFS), DMFS, and OS rates were 80, 90, and 92% in the ICT group, respectively. The most frequent grade 3/4 toxicities in 239 patients treated with ICT plus CCRT were neutropenia (42%), leukopenia (41%), and stomatitis (41%). Another study conducted by Cao and co-workers (16) showed the 3-year DFS, OS, and DMFS rates were 82.0, 88.2, and 86.0%, respectively, in the ICT/CCRT arm, and the most frequent grade 3/4 toxicity was neutropenia (16.0%). Meanwhile, the study of Zhang et al (8). showed that the 3-year RFS, DMFS and OS rates were 85.3, 91.1, and 94.5% in the ICT arm, respectively, and the incidence rate of acute grade 3/4 adverse events was 75.7%. In the present study, the survival rates were relatively similar to those reported previously (6, 8, 16), but acute adverse events were more commonly found in the ICT followed by CCRT plus ACT group than without ACT group. Patients have been included till December 2018, consequently part of the cohort has a very short follow-up. Longer follow-up is needed to fully assess survival and late toxic effects.

Compared to ICT/CCRT arm, ICT followed by CCRT plus ACT did not seemingly improve treatment outcomes in patients with LA-NPC. The less beneficial effects of this regimen can be explained by the low adherence to treatment due to treatment-related toxicities (**Table 2**). Only 60 patients received at least two cycles of ICT, CCRT, and ACT each, and only 48 patients received at least three cycles of ICT and two cycles CCRT. Nine patients receiving ICT had dose reductions, while 34 patients receiving ACT had dose reductions. Another possible reason is that we analyzed the data without stratification. Thus, we subsequently performed subgroup analysis, and found that the 3-year DFS, OS, LRRFS, and DMFS rates were 89.2, 95.7, 92.7, and 96.5% for stage III LA-NPC, and 70.6, 83.3, 90.2, and 76.4% for stage IVA/IVB LA-NPC, respectively (**Figure 2**). A prospective phase 2 clinical trial on ICT followed by concurrent chemoradiation for LA-NPC showed that the 3-year DMFS, progression-free survival (PFS), local PFS, and OS rates were 100, 82.3, 94.2, and 96% for stage III NPC patients and 95.1, 98.1, 81.3, and 93.4% for stage IVA/IVB NPC patients, respectively (17). Thus, the treatment outcomes of ICT followed by CCRT plus ACT were not superior to those of ICT followed by CCRT in stage III or IVA/IVB patients. However, this conclusion needs to be interpreted with caution. Because four different chemotherapy regimens have been used in 163 patients in the current study, the cohort is heterogeneous with regard to the regimen of induction and adjuvant chemotherapy.

In addition, the 3-year DFS rates were 100, 87.4, 72.4, and 69.5%, respectively, for stage N0-3 LA-NPC patients; the 3-year OS rates were 100, 89.8, 89.6, and 87.8%, respectively, for stage N0-3 LA-NPC patients; and the 3-year DMFS rates were 100, 93.4, 81.3, and 75.5%, respectively, for stage N0-3 LA-NPC patients. Li et al (7). reported that ICT remarkably enhanced failure-free survival (FFS) and OS in LA-NPC patients. Although the survival benefits of ICT are mainly associated with the reduced distant metastases, these beneficial effects have only been noted in patients with N1 stage but not with N2 or N3 stage (7). A retrospective study reported by Xu et al (18). suggested that ACT might not possess additional beneficial effects on NPC patients with N2 stage, but could improve OS and reduce distant metastases in those with N3 stage. In this study, the 3-year DFS, OS, and DMFS rates were 69.5, 87.8, and 75.5%, respectively, for LA-NPC patients with stage N3, which are in good agreement with the findings of Xu’s study (18). The number of stage N3 LA-NPC patients in this study is relatively small (17 patients), hence, it is necessary to verify these results in the near future. Another study investigated the efficacy, feasibility, and safety of four cycles of ICT/CCRT in stage N3 NPC patients. The 3-year DMFS, OS, and PFS rates were 81.8, 90.9, and 81.8%, respectively, which seem to be superior to the survival rates of our patients. However, ICT followed by CCRT plus ACT was associated with low tolerance and high toxicity, thus reducing its effectiveness.

Optimization of the ACT regimen (by replacing 5-FU with tegafur) can reduce toxicities and improve treatment efficacy in NPC patients. Zhang and co-workers (19) demonstrated that CCRT plus S-1 ACT could decrease toxicity and enhance survival in NPC patients with N3 stage. The 3-year DMFS, OS, and PFS rates were 84.1, 86.4, and 81.8%, respectively. Zong et al (20). reported that the survival rates were better in stage N3 NPC patients treated with IMRT followed by maintenance chemotherapy using S-1 than in those treated with CCRT without maintenance chemotherapy, in which the 3-year DMFS (90.5 *versus* 70.3%, p < 0.05) and OS (95.2 *versus* 76.3%, p < 0.05) rates were comparatively higher. Nevertheless, some clinical trials are undergoing to evaluate the safety and effectiveness of oral medicine. Induction chemotherapy followed by concurrent chemoradiotherapy plus oral medicine-based adjuvant chemotherapy may be a choice for patients with LA-NPC, especially for patients with N3 disease in the future.

This retrospective analysis has several weaknesses. First, there was a lack of head-to-head comparison between ICT+CCRT group and ICT+CCRT+ACT group. The value of AC cannot be judged in the current study. Second, the heterogeneity of induction/adjuvant chemotherapy regimens may cause a selection bias. Third, the follow-up is very short in a subset of patients. Fourth, the sample size is limited. So, the outcomes of this study should be interpreted with caution.

## Conclusion

In summary, ICT followed by CCRT plus ACT did not seemingly enhance DFS and OS in patients with LA-NPC compared to the addition of ICT to CCRT (historical controls). In contrast, ICT followed by CCRT plus ACT had more acute adverse events than ICT followed by CCRT. Longer-term clinical studies are required to examine the treatment outcomes and late toxicities of this combined regimen.

## Data Availability Statement

The original contributions presented in the study are included in the article/supplementary material; further inquiries can be directed to the corresponding author.

## Ethics Statement

The studies involving human participants were reviewed and approved by the ethics committee of the Second Affiliated Hospital of Nanchang University. The patients/participants provided their written informed consent to participate in this study.

## Author Contributions

J-WD was responsible for the thesis guidance. LZ and QL were responsible for the project design and paper writing. RZ and J-JY were responsible for the data collection. J-LH was responsible for the statistical analysis. Z-WT has contributed to article writing and paper revised. All authors contributed to the article and approved the submitted version.

## Funding

This research was funded, in part, by the Natural Science Foundation of China (81660452 and 81660453), Project of Health Commission of Jiangxi Province (20191093), and the Jiangxi Natural Science Foundation of China (20202BAB206057).

## Conflict of Interest

The authors declare that the research was conducted in the absence of any commercial or financial relationships that could be construed as a potential conflict of interest.
